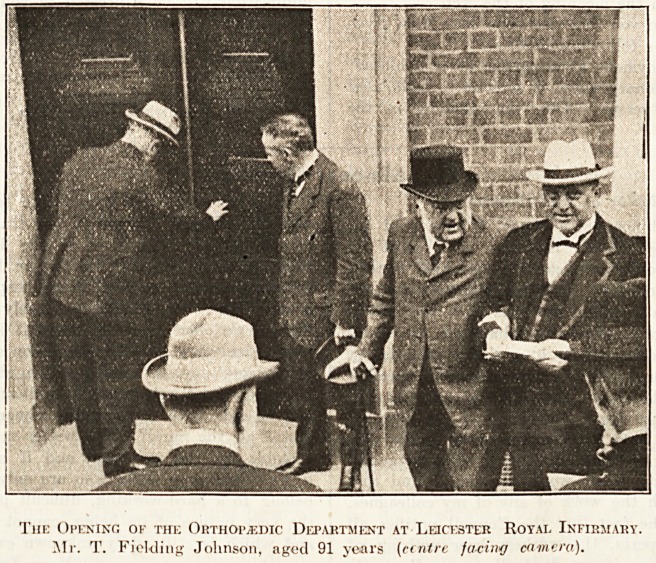# Fielding Johnson of Leicester

**Published:** 1921-03-26

**Authors:** 


### A FAMILY'S GREAT RECORD.

### Fielding Johnson of Leicester.

THE death on March 18 of Mr. T. Fielding Johnson, J.P., of Leicester, at the age of ninety-two years, removes a citizen who played an important part in the life and well-being of the city. He was the oldest Governor of the Leicester Royal Infirmary, the Senior Vice-President, and Trustee. He became a yearly subscriber in the year 1861, and from that time his interest has remained constant.

During the long Chairmanship of the late Sir Arthur Hazlerigg, Bart., from 1863 to 1890, Mr. Fielding Johnson was an active member of the weekly Board. His residence in Leicester enabled him to give considerable time to the administration of the Infirmary, so that for many years, by reason of the knowledge he possessed of the actual working, he was regarded by the two Chairmen under whom he served as the administrative Chairman of the institution. He was appointed Chairman in 1888. Under his auspices a beginning was made with the great scheme of development, which has continued until the present day. This first addition took the form of a new wing, with operating theatres, etc., and formed part of the local memorial to Queen Victoria's beneficent reign in the year 1900. In 1902, to the deep regret of the Governors, Mr. Fielding Johnson resigned the Chairmanship; he, however, continued to act as Chairman of the Trustees of the Sutton Charity, which makes monetary grants to deserving in-patients of the institution, and with unfailing regularity he attended at the infirmary each Saturday in discharge of the duties of the Trust.

THE OPENING OF THE ORTHOPÆDIC DEPARTMENT AT LEICESTER ROYAL INFIRMARY. Mr. T. Fielding Johnson, aged 91 years (*centre facing camera*).

Mrs. Fielding Johnson, who predeceased him, closely associated herself with her husband's work for the infirmary, and on Sir Edward Wood undertaking the Chairmanship she acceded to his invitation to become the first lady member of the Board. Throughout Sir Edward Wood's Chairmanship Mr. and Mrs. Fielding Johnson gave encouragement to the Board in the building developments, and were generous contributors to the building funds.

A tablet in one of the new children's wards hands down to posterity the following record:—

THE "FIELDING JOHNSON" WARD.
NAMED IN RECOGNITION OF
THE GREAT SERVICES OF
MR. AND MRS. T. FIELDING JOHNSON.
M.C.M.XIII.

But the service of the Fielding Johnson family is not ended, and it proved a happy choice that the Board selected Mr. T. Fielding Johnson, Jun., as Chairman, in the year 1916, a position he still holds. Numerous additions and improvements have been completed under his direction, and at the present time a new wing of 100 beds and an enlarged Nurses' Home for sixty-six additional nurses are being constructed at a cost of £70,000. One of these additions was a new orthopædic department, which was opened in July last. Mr. Fielding Johnson senior was an interested spectator; four generations of the family were that day present. The youngest, Miss Elizabeth Everard, aged two years, handed to the Treasurer a cheque for £2,000 contributed by the present Chairman to open the department free of debt.

WORK FOR THE CITY.

The interest of Mr. Fielding Johnson senior in the welfare of the city was marked by many acts of generosity, the last and greatest being the munificent gift of the site for a university college and for the Wyggeston Schools. This site, on which the 5th Northern General Hospital was erected, he purchased from the county authorities eighteen months ago. Mr. Fielding Johnson was a Freeman of the City, and its oldest magistrate, and on the occasion of the visit of the King and Queen in the year 1919 he had the honour of being presented to their Majesties as "Leicester's Grand Old Man."

## Figures and Tables

**Figure f1:**